# Clinicopathological Characteristics and Outcomes of Genitourinary Rhabdomyosarcoma in Two Girls

**DOI:** 10.1002/ccr3.72691

**Published:** 2026-05-10

**Authors:** George Evele, Francine Kouya, George Ngock, Richard Bardin

**Affiliations:** ^1^ Paediatric Oncology Unit, Mbingo Baptist Hospital Mbingo Cameroon; ^2^ Paediatric Surgery Unit, Mbingo Baptist Hospital Mbingo Cameroon; ^3^ Pathology Unit, Mbingo Baptist Hospital Mbingo Cameroon

**Keywords:** chemotherapy, embryonal subtype, genitourinary rhabdomyosarcoma, urinary tract infection, vulvar swelling

## Abstract

Rhabdomyosarcoma (RMS) is a soft tissue neoplasm accounting for about 8% of solid tumors in children. There are mainly four histologic subtypes of RMS: embryonal, alveolar, spindle cell/sclerosing, and pleomorphic. The genitourinary tract is the second most affected primary site of RMS. Genitourinary RMS is more common in young boys than girls. Among the subtypes of RMS, embryonal RMS is the primary histologic subtype identified in the bladder and female genitourinary tract of children. We present two cases of genitourinary RMS in two girls, one affecting the bladder/urethra and the other affecting the vulva, both with the same embryonal histology. The first case presented initially with nonspecific symptoms of recurrent urinary tract infection before the development of a vulvar mass. The second patient initially presented with a vulvar swelling that could be misdiagnosed as an imperforate hymen. The tumor in the first patient exhibited a high mitotic count and demonstrated a positive response to chemotherapy. In contrast, the second patient's tumor presented with a low mitotic count and exhibited a poor response to chemotherapy. Although multimodal treatment could have improved the outcome for the first patient, radiotherapy could not be afforded as an adjunct after surgical intervention. Upfront surgery may have been more effective than neoadjuvant chemotherapy for the second patient, whose tumor had a low mitotic count. In low‐ and middle‐income countries, the prognosis for children diagnosed with soft tissue sarcoma remains unfavorable, attributable to a variety of multifactorial causes. Timely diagnosis and implementation of cost‐effective multimodal treatments can significantly improve outcomes in children with genitourinary rhabdomyosarcoma.

## Key Clinical Message

Rhabdomyosarcoma is the most common soft tissue sarcoma in the genitourinary tract of girls. A pelvic ultrasound is recommended in young girls with recurring urinary symptoms or vaginal bleeding. Clinical outcomes improve with multimodal treatment, but limited access to affordable pelvic radiotherapy in developing countries leads to poorer outcomes.

## 1 Introduction

Rhabdomyosarcoma (RMS) is a malignant soft tissue neoplasm [1]. RMS represents about 40% to 50% of all soft tissue sarcomas and 8% of solid malignancies in children [2, 3]. It displays a bimodal age distribution, with a higher prevalence in preschoolers compared to adolescents [2]. RMS is thought to primarily originate from mesenchymal cells, which are programmed to differentiate into striated muscle [1]. However, RMS can develop in organs with or without striated muscle throughout the body [1]. Aberrant activation of the hedgehog pathway in non‐myogenic endothelial progenitor cells can lead to the development of RMS in organs void of striated muscle [4]. There are four histologic subtypes of RMS, namely embryonal, alveolar, spindle cell/sclerosing, and pleomorphic [1].

The genitourinary tract is the second most affected primary site of RMS after the head and neck [1]. Other affected sites include the trunk, retroperitoneum, liver, salivary glands, biliary tract, gallbladder, and extremities [1, 4, 5]. Genitourinary rhabdomyosarcomas (RMSs) are typically categorized into bladder/prostate and non‐bladder/prostate types, with the latter group generally having more favorable outcomes [5]. The female genital tract RMS comprises about 3.5% of pediatric RMS cases and about 90% of those affecting the vulvo‐vaginal region [6]. Embryonal RMS is the primary histologic subtype found in the bladder and female genital tract, with the botryoidal embryonal variant being the most prevalent type in children [5]. The clinical presentation of genitourinary RMS depends on the primary anatomic site and the clinical stage of the disease [1]. Symptoms can be categorized as specific or nonspecific [1]. Nonspecific genitourinary symptoms include dysuria, hematuria, urine retention, vaginal bleeding, and pelvic pain [1]. A common specific symptom is a vulvovaginal mass, often described as resembling a “bunch of grapes” [1]. Consequently, constipation can be a secondary effect of the mass [5, 6]. The multimodal approach to managing genitourinary RMS has improved overall survival rates to over 80% in developed countries [7]. The lower overall survival rates of genitourinary RMS in developing countries can be attributed to a wide range of influential factors [8].

We present two cases of genitourinary rhabdomyosarcoma in two girls, aged 4 and 5, with a similar embryonal histological subtype; however, they exhibited different responses to treatment.

## 2 Cases

### 2.1 Case One: Clinical Presentation and Investigations

A 4‐year‐old girl who initially presented at a peripheral hospital with a history of frequent painful urination for about 3 weeks. It was associated with fever and lower abdominal pain. She was febrile with significant suprapubic tenderness. A presumptive diagnosis of urinary tract infection was made. A urinalysis showed the presence of 30–40 white blood cells (WBCs) and 8–12 red blood cells (RBCs) per high‐power field (HPF). She received empiric antibiotics and analgesics. Two weeks later, she remained afebrile but experienced worsening painful urination and constipation. A repeat urinalysis revealed motile bacteria, positive nitrite, over 100 WBCs/HPF, and 10–15 RBCs/HPF. She was admitted and received ceftriaxone for about a week. A urine culture revealed *Escherichia coli* , sensitive only to imipenem and amikacin. Symptoms resolved after a 7‐day course of imipenem.

However, 10 days after her last admission, she presented with a protruding swelling from the vulva that the mother had noticed 4 days before the consultation. The vulvar swelling was rapidly increasing in size and was associated with intermittent mild bleeding and vulvar itching. She had no prior history of cancer and no family history of cancer among her siblings.

She was referred to see a pediatrician whose clinical examination confirmed a mass originating from the urethra. The urethral mass measured approximately 5.0 × 6.0 cm, was pink in color, firm, fixed, and bled upon contact. An abdomino‐pelvic ultrasound showed an irregular anterior‐inferior, isoechoic solid bladder mass attached to the anterior wall. The mass measured approximately 3.6 × 1.5 × 3.6 cm. There was mild bilateral hydronephrosis; however, the renal function remained normal.

The patient was then referred to our pediatric surgical clinic. The following were performed: vaginoscopy, cystoscopy, and biopsy of the urethral mass performed under anesthesia. The cystoscopy showed multiple polyp‐like masses in the urethra, while the vaginoscopy results were benign.

**FIGURE 1:**
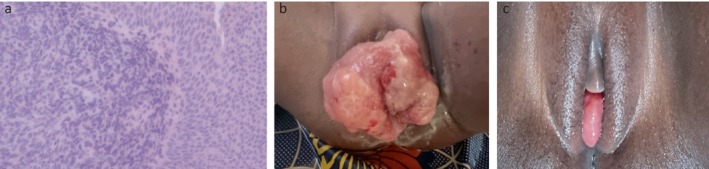
(a) Spindle cell neoplasm arising from the urothelium with a cambium pattern. (b) Cauliflower‐like vulva mass before chemotherapy. (c) Protruding peanut‐sized vulva mass after chemotherapy.

The histopathological analysis revealed a polypoid soft tissue fragment, white to tan in color, measuring 1.5 × 1.3 × 1 cm. Also, the sections revealed tissue composed of cells with bluntly spindled, round to irregular, hyperchromatic nuclei (Figure 1a). Cytoplasmic borders were not discernible. The background was eosinophilic. Cellularity varies from moderate to high. There was frequently a prominent neutrophilic infiltrate. The surface layer was urothelium. The mitotic rate was 12/10 HPFs, and no necrosis was seen. There was the appearance of a cambium layer focally, but this is not displayed broadly. Tumor was present throughout the tissue to the edges of the sections. These findings were suggestive of a spindle cell neoplasm favoring embryonal rhabdomyosarcoma (ERMS), and with positive margins.

Unfortunately, the child was lost to follow‐up, and all efforts to contact the parents were unsuccessful. The patient presented at our pediatric oncology clinic 6 months later with a huge painful swelling protruding through the vulva associated with lower urinary tract symptoms (Figure 1b).

She was admitted, and investigations revealed leukocytosis (13,810 cells/mm^3^), severe microcytic hypochromic anemia (hemoglobin 5.0 g/dL), and marked thrombocytosis (1033 cells/mm^3^). The metabolic panel showed mildly elevated potassium (5.9 mmol/L) and creatinine (1.24 mg/dL). Urine culture isolated *Acinetobacter baumannii* , sensitive only to doxycycline. Chest X‐ray was not suggestive of metastatic lesions, while abdominal ultrasound indicated obstructive uropathy and a bladder wall mass, with no liver or retroperitoneal masses. She was transfused and her urinary tract infection treated. According to the Children's Oncology Group (COG) pretreatment clinical staging for RMS, the patient was classified as stage 3 due to an unfavorable primary tumor and an invasive tumor size greater than 5 cm. Additionally, the patient was assigned to COG's clinical group III because of incomplete resection with gross residual disease after biopsy. A combination of the COG clinical stage and clinical group resulted in a risk stratification classified as intermediate risk. This was an approximate clinical staging; bone marrow involvement was not ruled out, FOXO1 (Forkhead box protein O1) fusion status was not determined due to capacity limitations, and less sensitive imaging modalities were used for clinical staging. In addition, the Ki‐67 proliferation index was not assessed.

### 2.2 Treatment

She received the VAC (vincristine, actinomycin, and cyclophosphamide) protocol, which included alternating combinations of vincristine at 1.5 mg/m^2^, actinomycin D at 1.5 mg/m^2^, and cyclophosphamide at 1 g/m^2^. She had four cycles of chemotherapy. The vulva mass had a more than 75% clinical reduction with chemotherapy (Figure 1c). During chemotherapy, she had grade 3 neutropenia and grade 2 unilateral ophthalmic herpes zoster, which were managed without complications.

### 2.3 Follow‐Up and Outcome

The preoperative evaluation found that clear margins were unattainable, so adjuvant radiotherapy was recommended. However, the family could not afford the cost of radiotherapy, so neither radiotherapy nor surgery was done. She received palliative care focused on pain management. The disease progressed, and she died in July 2024.

### 2.4 Case Two: Clinical Presentation and Investigations

**FIGURE 2:**
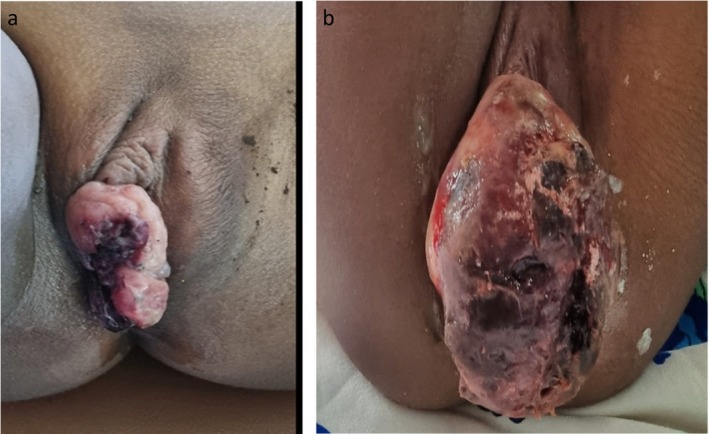
(a) Vulva mass with a necrotic central region before chemotherapy. (b) Profound increase in the size of the vulva mass after one cycle of chemotherapy.

A 5‐year‐old girl presented at our pediatric oncology clinic with a painful vulva mass for 3 months. The guardian noticed a painless swelling bulging through the vaginal opening while bathing the patient. She consulted the child at a hospital where an imperforate hymen and a tumoral process were suspected. She did not have any prior history of cancer, nor was there a family history of malignancy. An excisional biopsy of the vulva mass was performed in January 2023. The histopathology report showed a proliferation of obviously dense neoplastic cells, with round cells, sometimes elongated, with scant cytoplasm and an oval or spindle‐shaped nucleus, blue in appearance. The cytoplasm had an eosinophilic and rhabdoid appearance. The mitotic activity was low. These observations were in favor of an ERMS originating from the vulva. The guardian declined medical management but resorted to complementary medicine. There was no improvement, and the vulva swelling increased in size from a peanut to the size of a grapefruit (Figure 2a). This prompted another consultation. A computed tomography scan of the chest, abdomen, and pelvis revealed local disease but no distant spread. According to the Children's Oncology Group (COG) clinical staging system of RMS, the patient was classified as clinical stage 1 due to a tumor size of less than 5 cm and a favorable primary site. Additionally, the patient was categorized into clinical group III because of the presence of gross residual disease after biopsy, leading to an intermediate risk stratification. This staging was approximate, as other factors such as bone marrow involvement and FOXO1 status were not assessed. Also, the Ki‐67 proliferation index was not assessed.

### 2.5 Treatment and Outcome

The multidisciplinary team decided to proceed with neoadjuvant chemotherapy using the VAI protocol (vincristine, actinomycin D, and ifosfamide) with granulocyte colony‐stimulating factor support and mesna.

She received one cycle, and the vulvar mass increased significantly to an orange‐sized mass over the next 3 weeks (Figure 2b). The regimen was changed to VAC; she received one cycle. Despite undergoing chemotherapy, the vulvar mass continued to progress. Although surgery and subsequent radiotherapy were recommended, the guardians, feeling disheartened by the chemotherapy's poor results, decided to discontinue all care.

## 3 Discussion

ERMS is the most common subtype of rhabdomyosarcoma (RMS), representing a significant 70% to 80% of all diagnosed cases [1]. About 25% of patients with ERMS receive their diagnosis within the crucial first 5 years of life, highlighting the importance of early detection and intervention [1]. The ERMS subtype represents approximately 80% of diagnosed histologic subtypes of RMS, primarily affecting the genitourinary tract [5]. In this light, the two girls presented were diagnosed with ERMS affecting the genitourinary tract within the first 5 years of life. The ERMS is composed of oval to spindled cells. The botryoidal variant of ERMS is commonly found in the bladder and vagina, displaying dense clusters of polypoid cells that, upon macroscopic examination, resemble a “bunch of grapes.” [1, 5]. Also, the botryoidal variant, which was observed in case one, demonstrates a distinctive feature of a cambium layer of cells, which is a condensation of tumoral cells underlying epithelial cells [1]. A classic pattern of ERMS was observed in case one, manifesting as areas of hypercellularity alternating with hypocellularity [1]. Alveolar RMS (ARMS) makes up about 20% of all RMS cases, and the anatomic sites of preference are the extremities, head and neck, perineum, and paraspinal regions [1]. Morphologically, ARMS shows a uniform round cell appearance, with the cells typically arranged in either a solid or alveolar pattern [1]. The spindle cell/sclerosing and pleomorphic RMSs are rarer subtypes with high‐grade features [1].

The most reliable method for diagnosing RMS is through the analysis of a tissue sample [1]. The tumors of some patients present with poorly differentiated morphology, which challenges traditional histopathological analysis [1]. However, the accuracy of diagnosis improves with immunohistochemical and molecular analyses [1]. Some immunohistochemical tumor markers for striated muscle cell differentiation include desmin, myogenin, and myogenic differentiation factor 1 (MyoD1) [1]. These markers assist in diagnosing RMS when the histopathological analysis is unclear [1]. ERMS shows patches of myogenin staining, while ARMS exhibits strong and diffuse myogenin expression [1]. The spindle cell/sclerosing RMS demonstrates a strong and diffuse expression of MyoD1, but shows faint staining for both desmin and myogenin [1]. In contrast, pleomorphic RMS exhibits strong positivity for desmin, while staining weakly for both MyoD1 and myogenin [1, 4]. The two girls were diagnosed with ERMS primarily through histopathological analysis using eosin and hematoxylin staining, without additional immunohistochemical methods.

ARMS features two genetic rearrangements that hold diagnostic and prognostic importance [7]. The genotypes include the PAX3‐FOXO1 gene fusion identified by t(2;13) (q35;14) and the PAX7‐FOXO1 gene fusion characterized by t(1;13) (p36;q14) [9]. FOXO1 fusion status is associated with a poor prognosis and should be tested for risk stratification [1, 3]. Our pathology unit does not perform cytogenetic studies, but FOXO1 status is mainly relevant for ARMS cases, whereas our patients had the ERMS subtype.

The exact causes of RMS remain elusive [1]. Although RMS is not a monogenic disease, certain sporadic driver mutations in somatic cells and some germline mutations associated with cancer predisposition syndromes increase the risk of developing RMS [3]. Autosomal dominant cancer predisposition syndromes include Li‐Fraumeni syndrome (TP53 mutations), retinoblastoma, and DICER1 syndrome [1]. RASopathies, resulting from mutations in the RAS (Rat Sarcoma) pathway, include Costello syndrome, Neurofibromatosis Type 1, and Noonan syndrome [3]. Beckwith–Wiedemann syndrome, linked to epigenetic changes on chromosome 11, is associated with an increased risk of RMS [3, 5]. Children with familial cancer syndromes usually develop RMS earlier in life and are often linked to the ERMS subtype in most cases [1, 3]. The two cases presented did not have cytogenetic studies to rule out familial cancer syndromes; however, there was no family history of cancer or noticeable dysmorphic features in either patient.

The standard of care in managing genitourinary RMS is a multidisciplinary approach [7]. This approach considers the significant genetic changes in the patient, the clinical stages ranging from stage 1 to stage 4, the clinical groupings from group I to group IV, and the anatomical site categorized as either favorable or unfavorable [7]. The clinical stage and group are used to risk‐stratify patients into categories that inform the intensity of treatment [5, 7]. The Children's Oncology Group has three risk groups: low, intermediate, and high [7]. The multimodal approach incorporates neoadjuvant chemotherapy to improve surgical success and downstaging of the disease. This strategy enhances local control by often incorporating delayed surgery or radiotherapy for intermediate‐risk patients, ultimately aiming to improve outcomes and reduce the risk of recurrence [7, 10]. The standard chemotherapy regimen for the management of RMS is either a combination of vincristine, actinomycin D, and cyclophosphamide (VAC) or vincristine, actinomycin D, and ifosfamide (VAI) [10]. Both cases presented were intermediate risk; the first case received VAC primarily, and the second case received VAI initially and then VAC. The chemotherapy response varied, even though both were of the same EMRS subtype; case one had a better response compared to case two. The plausible differences in response may stem from the difference in anatomic site and level of mitotic count. Case one had an RMS located in the bladder, an unfavorable site, with an intermediate mitotic count. In contrast, case two had RMS in the lower female genital tract, a favorable site, but with a low mitotic count. Mitotic count has been associated with playing a role in chemotherapy response; neoplasms with low mitotic counts typically have poor responses [11]. Relapse is a common problem in patients with RMS, especially those with positive surgical margins; however, recurrence rates are lower with the use of combined chemotherapy and radiotherapy [7].

Rhabdomyosarcoma (RMS) can spread to the lungs, liver, bones or bone marrow, as well as distant lymph nodes [12]. The risk of metastasis in localized genitourinary RMS cases is low [7]. However, research shows that even in patients with localized RMS, tumor cells can be detected in peripheral blood and bone marrow [13]. Thus, bone marrow sampling is essential in children diagnosed with RMS for proper staging [12]. However, the two cases presented did not undergo bone marrow analyses. ARMS has a higher tendency for metastasis compared to other RMS subtypes, with about 25%–30% of patients diagnosed with metastatic disease [1]. In RMS cases with bone marrow involvement, clinical presentation can mimic symptoms of leukemia [1, 5]. ARMS and a positive FOXO1 fusion status are associated with a poor prognosis [1]. In resource‐limited settings, losing patients to follow‐up is a common challenge that negatively impacts prognosis and requires appropriate interventions to mitigate it [14]. Oberlin et al. [12] identified several risk factors commonly known as Oberlin risk factors that are associated with a worse prognosis in children with RMS: age below 1 year or above 10 years, an unfavorable primary tumor location, the presence of three or more metastatic sites, and involvement of the bone marrow. Unlike nephroblastoma, where anaplasia serves as a poor prognostic marker, it holds no prognostic value in RMS [1, 15]. In high‐income countries, the overall survival for children with genitourinary RMS exceeds 80%, while low‐ and middle‐income countries experience much lower rates [1, 7]. Early diagnosis, standard chemotherapy regimens, timely organ‐conserving surgeries, and radiotherapy contribute to these better outcomes [2, 7]. Although pelvic radiotherapy could have improved the outcome for both cases, unfortunately, neither benefited from this vital modality of treatment for genitourinary RMS [16].

## 4 Conclusion

Genitourinary rhabdomyosarcoma in young girls can lead to significant morbidity and mortality, particularly in developing countries. In the initial stage of the disease, it may present with nonspecific symptoms that resemble those of a urinary tract infection. Risk stratification is based on the anatomic site of the tumor, which is crucial for effective management. Although multimodal treatment improves outcomes for patients with rhabdomyosarcoma, such services may not be accessible or affordable in developing nations. Upfront surgery should be considered in those with localized disease associated with low mitotic activity on histology. The Ki‐67 proliferation index offers a more effective approach to predictive and prognostic management in genitourinary RMS compared to the traditional mitotic count. Its significance should be recognized and emphasized before chemotherapy in patients with genitourinary RMS.

## Author Contributions

**George Evele:** conceptualization, data curation, investigation, methodology, supervision, visualization, writing – original draft, writing – review and editing. **Francine Kouya:** methodology, writing – review and editing. **George Ngock:** investigation, writing – review and editing. **Richard Bardin:** investigation, supervision, validation, writing – review and editing.

## Funding

The authors have nothing to report.

## Consent

The parents of both patients gave written informed consent for the study and images for this publication.

## Conflicts of Interest

The authors declare no conflicts of interest.

## Data Availability

The consent forms are available upon request.
